# Cellulose Nanocrystals Derived from Textile Waste through Acid Hydrolysis and Oxidation as Reinforcing Agent of Soy Protein Film

**DOI:** 10.3390/polym12040958

**Published:** 2020-04-20

**Authors:** Shuting Huang, Ran Tao, Ashraf Ismail, Yixiang Wang

**Affiliations:** Department of Food Science and Agricultural Chemistry, McGill University, Ste Anne de Bellevue, QC H9X 3V9, Canada; shuting.huang@mail.mcgill.ca (S.H.); ran.tao5@mail.mcgill.ca (R.T.); ashraf.ismail@mcgill.ca (A.I.)

**Keywords:** cellulose nanocrystals, textile waste, acid hydrolysis, three-step oxidization, reinforcing agent

## Abstract

More than 10 million tons of textile waste are disposed through landfill every year in North America. The disposal of textile waste via landfill or incineration causes environmental problems and represents a waste of useful resources. In this work, we explored the possibility to directly extract cellulose nanocrystals (CNCs) from untreated textile waste through two methods, namely sulfuric acid hydrolysis and three-step oxidization. CNCs with cellulose *I_β_* crystalline structure and rod-like shape were successfully obtained. The aspect ratios of CNCs prepared from acid hydrolysis and oxidization were 10.00 ± 3.39 and 17.10 ± 12.85, respectively. Their application as reinforcing agent of soybean protein isolate (SPI) film was evaluated. With the addition of 20% CNCs, the composite film maintained the high transparency, while their water vapor barrier property, tensile strength, and Young’s modulus were significantly improved. This research demonstrates a promising approach to recycle textile waste, and more value-added applications based on the derived CNCs could be expected.

## 1. Introduction

Today, there is a concern about the rapid increase in textile waste, which leads to numerous disposal problems and governance issues [1]. According to the statement of Waste Reduction Week in Canada [2], textile waste has shown a dramatic increase in the past two decades, and about 10 million tons of clothing are disposed of through landfill every year. It was reported that only 20% of post-consumer clothes were recycled, but without appropriate processing [3]. Therefore, in one aspect, some efforts, such as implementing circular economy principles, are needed to reduce textile waste and achieve sustainable development; on the other hand, it is necessary to find a promising approach to recycle textile waste into valuable products. For instance, Çay et al. converted textile waste into biochar by low-temperature carbonization, and applied the derived biochar to cotton fabrics, which possessed the odor masking function and high thermo-physiological comfort [4]. Besides, Xu et al. derived char-based adsorbents from textile waste by a one-step low-temperature pyrolysis approach with iron salts, and the prepared adsorbents owned adequate adsorption capacity of Cr(VI) removal [5]. However, more efforts are required to promote the recycling of textile waste.

One of the most consumed fibers in the textile industry is cotton, which contains more than 90% of cellulose. Therefore, it was supposed that waste cotton fibers could act as the new source of cellulose nanocrystals (CNCs). CNCs have received considerable interest over the past decades owing to their unique mechanical properties. They possess high crystallinity and large specific surface area [6]. Additionally, CNCs contain abundant hydroxyl groups, contributing to the high potential for modifications and applications. CNCs could be extracted from three types of raw materials: plant, animal, and microorganism, where sulfuric acid (H_2_SO_4_) hydrolysis is the most frequently used method [7]. For instance, Favier et al. successfully used acid hydrolysis to extract CNCs from tunicate and applied them to reinforce polymer nanocomposites [8]. Darpentigny et al. also obtained CNCs with a high aspect ratio of 63 from tunicate by H_2_SO_4_ hydrolysis, and the prepared tunicate CNC-based cryogels presented high mechanical resistance and stiffness (Young’s modulus of 138 KPa) [9]. Wood was another common raw material of CNCs. The rod-shaped CNCs with 11.4 nm diameter and 153.2 nm length were obtained from wood via acid hydrolysis [10]. Furthermore, Ambrosio-Martin et al. obtained CNCs, with a length of around 600 nm and diameter of around 22 nm, from bacteria through H_2_SO_4_ hydrolysis, which were used to improve the barrier and mechanical properties of polylactide (PLA) [11]. Recently, a new trend has been found to prepare CNCs from waste materials. For example, Jiang et al. successfully extracted CNCs from wastepaper fibers by acid hydrolysis. The obtained CNCs had a rod-like structure with the crystallinity of 77.56% [12]. Similar rod-like CNCs were extracted from the disposed paper cup through citric acid hydrolysis and were applied to reinforce composite films [13].

In addition to acid hydrolysis, oxidation methods have been developed recently, which could introduce anionic groups to CNCs for better performance [14,15,16]. Leung et al. developed an approach to prepare carboxylic CNCs using ammonium persulfate as oxidant. The results indicated that the presence of carboxylic acid groups on CNC surface provided active sites for further modification, such as protein/enzyme immobilization. Additionally, carboxylic CNC offered an opportunity for tuning hydrophobicity by conjugation of the carboxyl groups with pertinent molecules [17]. TEMPO, (2,2,6,6-Tetramethylpiperidin-1-yl)oxyl, is another widely used radical for oxidizing CNCs, which could selectively oxidize the primary alcohol groups of CNCs into carboxyl groups. For example, Liu et al. used TEMPO-mediated oxidation to prepare CNCs with a rod-like morphology (diameter of 2–4 nm and length of 20–50 nm), higher surface charge, and better redispersibility [18]. Recently, a three-step oxidation method has been developed to introduce more anionic groups for better dispersability and further modifications. The first step was performed by periodate oxidation, resulting in partial 2.3-dialdehyde CNCs. The dialdehyde CNCs were then treated with chlorite oxidation to converse dialdehyde groups into dicarboxylic groups. The third step was TEMPO oxidation, and the C-6 hydroxyl groups were oxidized into carboxyl groups [19].

It was noticed that Wang et al. reported the extraction of CNCs from waste cotton cloth by a series of pre-treatments and a mixed acid solution [20]. However, the application of the obtained CNCs has not been evaluated. Therefore, the current work aims to explore the possibility to directly extract CNCs from untreated textile waste through two different methods, namely sulfuric acid hydrolysis and three-step oxidization. The morphology and structure of CNCs were characterized by transmission electron microscopy (TEM), Fourier-transform infrared spectroscopy (FT-IR), and X-ray diffraction (XRD). Moreover, soy protein isolate (SPI) was selected as the model of biodegradable materials. SPI is the major component of soybean, and various applications such as adhesives, plastics, and binders have been suggested in recent years. However, SPI-based materials suffer from their poor water resistance and low strength [21]. Therefore, the effects of CNCs derived from textile waste on the optical, water vapor barrier, and mechanical properties of SPI films were studied.

## 2. Materials and Methods

### 2.1. Materials

Waste cotton clothes were kindly provided by Renaissance (Montreal, QC Canada), and soybean protein isolate (SPI) was provided by Cargill (Minneapolis, MN, USA). Hydrogen peroxide (H_2_O_2_), sodium dihydrogen phosphate (NaH_2_PO_4_), and disodium hydrogen phosphate (Na_2_HPO_4_) were purchased from Fisher Scientific (Ottawa, ON, Canada) and used as received. Reagent alcohol (95%) was obtained from the RICCA Chemical company (Arlington, VA, USA). Sulfuric acid (H_2_SO_4_), and sodium periodate (NaIO_4_), sodium chloride (NaCl), ethylene glycol, sodium chlorite (NaClO_2_), sodium hypochlorite (NaClO), sodium hydroxide (NaOH), glycerol, and (2,2,6,6-Tetramethylpiperidin-1-yl)oxyl (TEMPO) were purchased from Sigma-Aldrich (Oakville, ON, Canada) and used without further treatment.

### 2.2. Extraction of CNCs

**Sulfuric acid hydrolysis.** No chemical pretreatment was performed before extraction, and CNCs were separated by sulfuric acid hydrolysis as described by Ko et al. [22]. In brief, cotton clothes (5.00 g) were mixed with 60 wt % H_2_SO_4_ solution (100.00 g) and stirred for 1 h at 25 °C. Then, the suspension was diluted with 1 L cold water to stop the hydrolysis. CNCs were washed three times with distilled water followed by freeze-drying and coded as HCNCs.

**Three-step oxidization.** Oxidized CNCs were prepared via a modified method of Yang et al. [23]. Firstly, cotton clothes (3.00 g) were added to the solution of 3.33 g NaIO_4_, 19.50 g NaCl, and 300 mL distilled water. The mixture was stirred at 25 °C for 36 h under the prevention of light. After that, the reaction was ended by adding ethylene glycol to quench the residual periodate, and the oxidized fibers were washed thoroughly with distilled water by filtration. Secondly, the oxidized fibers were mixed with 3.56 g NaClO_2_, 14.60 g NaCl, 3.30 g H_2_O_2_, and 250 mL distilled water, and the mixture was stirred at 25 °C for 24 h. Then, the suspension was washed thoroughly with a water–ethanol solution and freeze-dried. Thirdly, 1.00 g of chlorite oxidized sample, 0.0016 g of TEMPO, 1.13 g of NaClO_2_, and 90 mL of phosphate buffer (pH 6.8) were mixed and stirred in the heated water bath. After the temperature reached 50 °C, 250 μL NaClO (diluted with 10mL phosphate buffer) was added to the mixture. The translucent suspension was obtained after 48 h, followed by washing with a water-ethanol solution and freeze-drying. The resultant sample was coded as TCNCs. The yield (%) of CNCs was calculated using a gravimetric method in terms of Equation (1):

(1) $$\mathrm{Yield}=\frac{w_{2}}{w_{1}}$$

where w_2_ is the weight of freeze-dried CNCs, and w_1_ is the weight of cotton clothes [24].

### 2.3. Preparation of CNC/SPI Films

**Figure 1:**
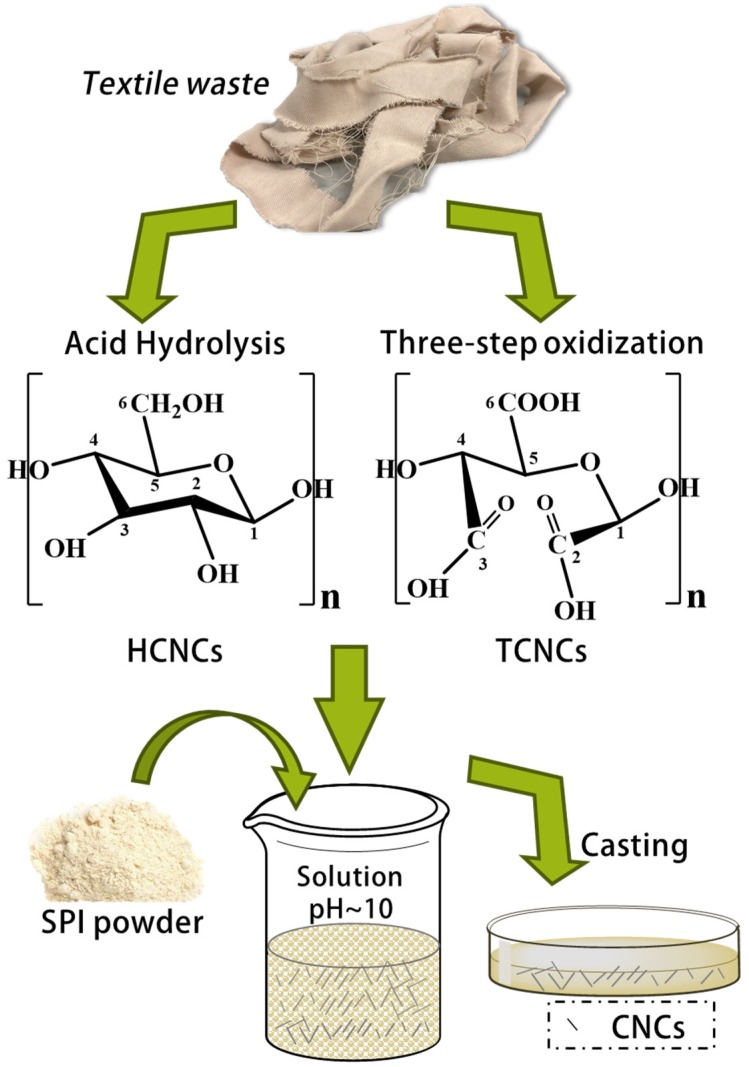
Schematic illustration of soy protein isolate (SPI)/cellulose nanocrystal (CNC) composite film preparation.

Soy protein isolate (SPI) films containing HCNCs and TCNCs were prepared by the solvent casting method [25], as illustrated in Figure 1. Briefly, 0.50 g of SPI was dissolved in 15 mL distilled water under magnetic stirring at 25 °C. Then, 0.30 g of glycerol (as the plasticizer) and 3 mL of NaOH solution (1 wt %) were added to the SPI solution, and the mixture was stirred for 15 min at 50 °C. After that, the desired amounts of CNCs (10% and 20% of SPI dry weight) were added and stirred overnight. The homogeneous solutions were cast on the plastic plate and dried at 25 °C for 24 h. SPI film without the addition of CNCs was prepared as control. All the samples were stored in the desiccator (~40% relative humidity (RH)) before further measurement [26].

### 2.4. Characterization

#### 2.4.1. Transmission Electron Microscopy (TEM) 

The morphology of HCNCs and TCNCs was observed on a transmission electron microscope (TEM, Morgagni 268, Philips-FEI, Hillsboro, OR, USA) at ×110,000 magnification. A small droplet of diluted CNC aqueous suspension was deposited on a polycarbon film supported on a copper grid, and a thin layer was suspended over the holes of the grid. The specimen was dried in air at ambient pressure, and was then imaged on TEM at an accelerating voltage of 80 kV. The dimensions of HCNCs and TCNCs were measured using the ImageJ image analysis software, which is developed by the National Institute of Health (version 1.52a, NIH, Bethesda, MD, USA).

#### 2.4.2. X-Ray Diffraction (XRD)

XRD patterns of HCNCs and TCNCs were collected using a Bruker D8 Discover diffractometer (Bruker, Billerica, MA, USA) operating at 40 kV and 40 mA, with a VANTEC detector and Cu-source. The crystallinity index (CrI) was determined by the peak height method [27] in terms of Equation (2):

(2) $$\mathrm{CrI}=\frac{I_{\left(200\right)}-I_{am}}{I_{\left(200\right)}}$$

where *I_(200)_* is the maximum diffraction intensity associated with surface areas of crystalline cellulose, and *I_am_* is the diffraction intensity of an amorphous cellulose fraction.

#### 2.4.3. Fourier-Transform Infrared Spectroscopy (FT-IR)

FT-IR spectra of HCNCs, TCNCs, and SPI/CNC films were obtained using the Varian Excalibur 3100 FT-IR spectrometer (Varian, Melbourne, Australia) equipped with an attenuated total reflectance (ATR) accessory (Specac, Orpington, UK). The spectra were collected as the average of 64 scans with a resolution of 4 cm^−1^ and 25 °C, using the empty accessory as blank. The measurements were carried out at 400–4000 cm^−1^ [28].

#### 2.4.4. Optical Transmittance Spectra

The optical transmittance of SPI/CNC films (the thickness was about 0.08 mm) was measured via a DU 800 UV/vis spectrophotometer (Beckman Coulter, Brea, CA, USA). The spectra were recoded with air as background.

#### 2.4.5. Water Vapor Permeability (WVP)

A gravimetric method was used to determine the WVP of SPI/CNC films [29]. In brief, anhydrous calcium chloride (15 g) was placed at the bottom of a glass jar. After sealing with film sample, the jar was placed in a desiccator filled with saturated sodium chloride solution (75% RH). The weight of the jar was measured periodically at 25 °C. Then, the WVP of SPI/CNC film was determined using Equation (3):

(3) $$\mathrm{WVP}=\frac{∆m\times n}{A\times t\times ∆p}$$

where *∆m* is the change in jar weight (g), *n* is the film thickness (m), *A* is the exposed area of the film (m^2^), *t* is the time (s), and *∆p* is the partial pressure difference existing between the two sides of film sample (Pa).

#### 2.4.6. Mechanical Properties

The tensile strength, elongation at break, and Young’s modulus of SPI/CNC films were tested on an eXpert 8612 biaxial testing machine (ADMET, Norwood, MA, USA) at 25 °C according to standard ASTM D882. The dimension of film specimen was 60 mm × 10 mm. The initial grip separation distance was set as 20 mm, and the separation speed was 20 mm/min.

#### 2.4.7. Statistical Analysis

The experimental data were presented as the mean of three batches ± SD (standard deviation) [30,31,32]. Analysis of variance (ANOVA) was applied for the statistical analysis followed by multiple comparison tests via Duncan’s multiple-range test, and differences within samples were identified at *p* < 0.05. All analyses were carried out using SPSS statistical software (version 24.0, IBM, New York, NY, USA).

## 3. Results and Discussion

**Figure 2:**
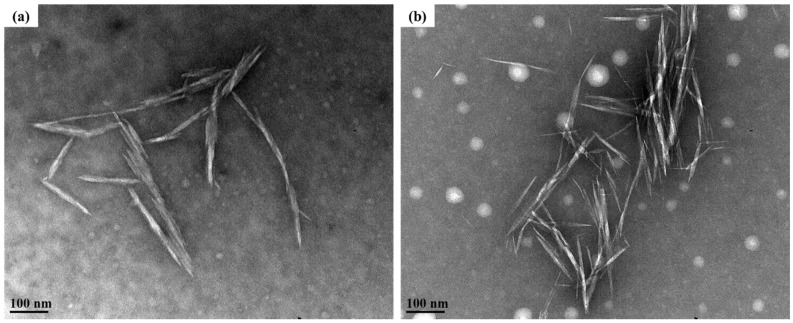
Transmission electron microscopy (TEM) images (×110,000 magnification) of HCNCs (**a**) and TCNCs (**b**).

The yields of HCNCs and TCNCs were 90.40% and 60.41%, respectively, which were comparable to those reported values [23,33,34,35,36]. Their morphology was observed by transmission electron microscopy (TEM). As shown in Figure 2, both of them had a typical rod-like shape, indicating the successful preparation of CNCs without any chemical pre-treatments. Particularly, the length and diameter of HCNCs were around 111.76 ± 38.73 nm and 11.18 ± 2.33 nm, and their aspect ratio was 10.00 ± 3.39. The TCNC samples presented a slightly shorter and thinner rod-like structure (diameter of 5.69 ± 2.08 and length of 97.25 ± 25.18, *p* > 0.05), which might be caused by the harsher reaction conditions during the three-step oxidation [37]. However, the aspect ratio of TCNCs was relatively higher (17.10 ± 12.85, *p* > 0.05). It was known that the higher aspect ratio of CNCs is conducive to a better reinforcing effect, as it increases the interface area and allows a higher load to be transferred within the crystal percolating network [38]. The aspect ratio of TCNCs was also higher than that of the CNC samples extracted from lettuce leaf [22] and comparable to those from cotton linters [39]. Therefore, it was demonstrated that different extraction methods had no significant effect on the morphology of CNCs, but contributed to the different aspect ratio values, which might affect their reinforcement efficiency in composite films [40].

**Figure 3:**
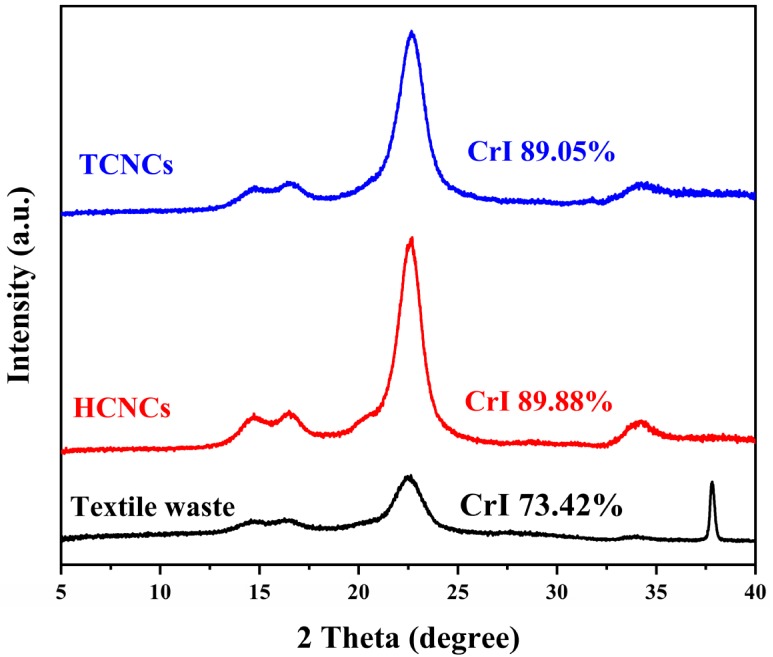
X-ray diffraction (XRD) patterns and crystallinity index (CrI) of textile waste, HCNCs, and TCNCs.

X-ray diffraction was used to evaluate the crystalline type and index of textile waste and CNCs. As shown in Figure 3, all the samples exhibited the same diffraction peaks at 15.0°, 16.4°, and 22.5°, corresponding to the (1**1**0), (110), and (200) lattice planes of cellulose *I_β_*, respectively [41]. This indicated that sulfuric acid hydrolysis and three-step oxidation did not alter the crystalline structure of cellulose [24]. However, the crystallinity index of TCNCs and HCNCs was 89.05% and 89.99%, respectively, which showed a remarkable increase compared with that of textile waste (73.42%). This was because the amorphous region of cotton fibers was effectively removed during CNC extraction.

**Figure 4:**
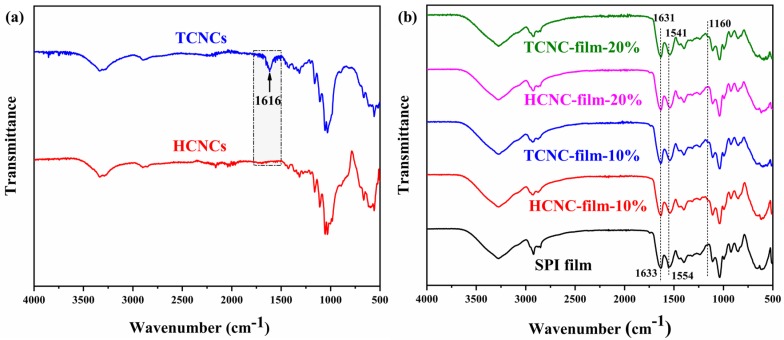
Fourier-transform infrared spectroscopy (FT-IR) spectra of (**a**) HCNCs and TCNCs, and (**b**) SPI and SPI/CNC films.

The FT-IR spectra of TCNCs and HCNCs are shown in Figure 4a. Both of them exhibited similar characteristic absorption peaks. For example, the broad peak at 3336 cm^−1^ was related to the stretching vibration of OH groups and the inter-chain hydrogen bonds [42]. The peak at 2900 cm^−1^ was attributed to the stretching vibration of C–H, and the peaks at 1427 cm^−1^, 1371 cm^−1^, and 1315 cm^−1^ were attributed to the bending of C–H, CH_2_, and OH, respectively, which were typical for polysaccharides. The peak at 1160 cm^−1^ was characteristic of the asymmetric vibration of (C–O–C), and those at 1055 cm^−1^ and 1031 cm^−1^ were associated with C–O–C pyranose ring (antisymmetric in phase ring) stretching vibration [43]. The peak at 893 cm^−1^ was characteristic of cellulose with β-glycoside bonds of glucose ring [36]. Especially, the characteristic peaks of cellulose *I_β_* appeared at 3290 and 3336 cm^−1^, and the peak at 1427 cm^−1^ is attributable to the crystalline absorption [44]. The absorbance ratio of the bands at 1427 and 893 cm^−1^ (*A_1427_*/*A_893_*), adopted as the crystallinity index, is closely related to the portion of the cellulose I structure [45]. Despite the use of different extraction methods, the ratios of *A_1427_*/*A_893_* were comparable, which indicated the similar crystallinity [46]. This result was in accordance with that of the XRD test. It was noticed that the only new peak of TCNCs existed at 1616 cm^−1^, which was assigned to the asymmetric vibration of –COO–. This indicated the successful extraction of CNCs with carboxyl groups by three-step oxidization [47].

Figure 4b displays the FT-IR spectra of SPI and SPI/CNC films. The peak at 2922 cm^−1^ was assigned to the C–H stretching, and the characteristic peak of cellulose appeared at 1160 cm^−1^. The main characteristic peaks of SPI were illustrated at 1633 cm^−1^, 1554 cm^−1^, and 1238 cm^−1^, presenting the Amide I (C–O stretching), Amide II (N–H bending), and Amide III (C–H and N–H stretching), respectively [48]. It was worth noting that the peaks of Amides I and Amide II showed a slight shift in terms of the CNC addition, indicating that there might be more exposed polar groups, and the bindings between the peptide chains increased. Li et al. suggested that the FT-IR peak shift at 1626 cm^−1^ and 1537 cm^−1^ with the addition of CNCs was the result of the molecular hydrogen bonding formed between protein and CNCs. That would result in an improvement in the mechanical properties [49]. There was no obvious change in the peak at 3275 cm^−1^. It was likely that some interactions among SPI were disrupted, and new bonding formed between SPI and CNCs, which were both reflected in FT-IR intensity [50].

**Figure 5:**
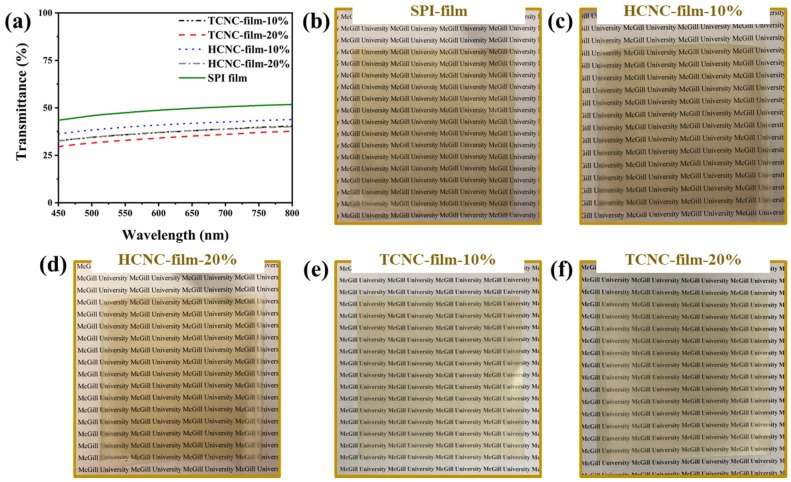
Optical transmittance (**a**) and photos (**b**–**f**) of SPI and SPI/CNC films.

Optical transmittance of SPI and SPI/CNC films is presented in Figure 5a. Evidently, the plain SPI film presented the highest transmittance among all the samples. The addition of CNCs into SPI film lessened its transparency, which decreased with the increasing amount of CNCs. This was because of the scattering or obstruction of light by the dispersed CNCs in SPI film, which was consistent with previous reports [51,52]. It was noted that, for the composite films containing the same content of CNCs, the transmittance at 600 nm of TCNC-film was higher than that of HCNC-film. This might be because of the better dispersion of TCNCs in water caused by the negatively charged carboxyl groups. However, despite the decreasing transmittance, the printed text under the films was still clearly visible, as shown in Figure 5b–f.

**Figure 6:**
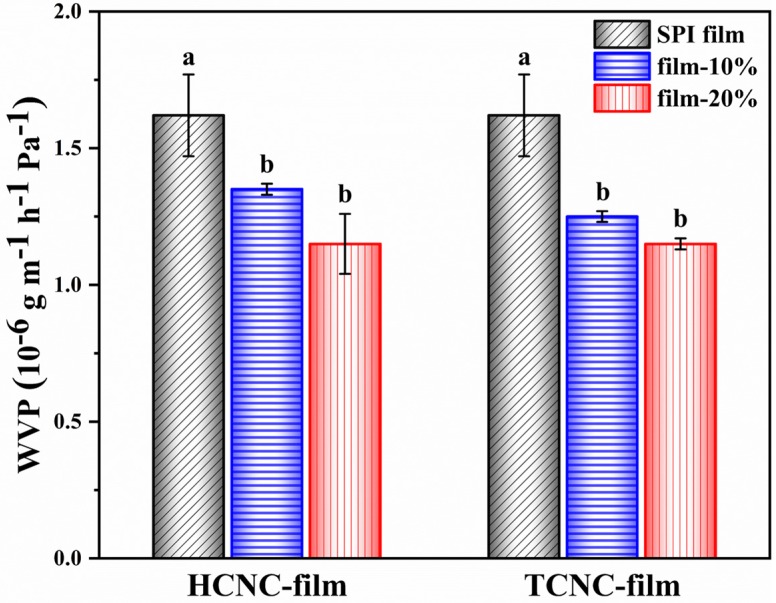
Water vapor permeability (WVP) of SPI and SPI/CNC films. Different letters on the tops of the columns indicate significant difference (*p* < 0.05) in terms of CNC content.

As depicted in Figure 6, the SPI/CNC films had an obviously lower WVP compared with the plain SPI film (*p* < 0.05). With the addition of 10% and 20% TCNCs, the WVP of SPI film significantly reduced from 1.62 ± 0.15 × 10^−6^ g m^−1^ h^−1^ Pa^−1^ to 1.25 ± 0.02 × 10^−6^ g m^−1^ h^−1^ Pa^−1^ and 1.16 ± 0.02 × 10^−6^ g m^−1^ h^−1^ Pa^−1^, respectively. A similar decrease in WVP values was also observed for HCNC-films. This indicated that both HCNCs and TCNCs derived from textile waste could enhance the water vapor barrier property of SPI film by decreasing the pathway of water molecules to transfer through the film [52]. However, the WVP differences between films containing 10% and 20% CNCs were not significant (*p* > 0.05).

**Figure 7:**
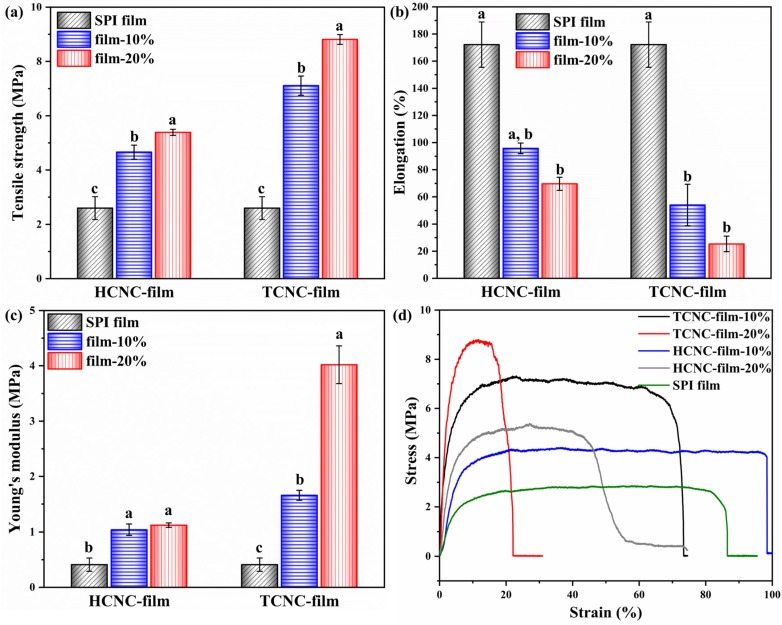
Tensile strength (**a**), elongation at break (**b**), Young’s modulus (**c**), and stress–strain curves (**d**) of SPI and SPI/CNC films. Different letters on the tops of the columns indicate significant difference (*p* < 0.05) in terms of CNC content.

The mechanical properties of SPI and SPI/CNC films were tested to evaluate the reinforcing effects of HCNCs and TCNCs derived from textile waste. As shown in Figure 7d, all the samples exhibited an initial linear elastic behavior followed by continuous growth in the slope on account of plastic deformation. After that, there was a plateau until the critical values of stress and strain were eventually reached, and the films broke [53]. It was worth noting that the addition of CNCs, both HCNCs and TCNCs, made an important contribution to mechanical properties. For example, the tensile strength of SPI films containing 10% and 20% HCNCs significantly increased from 2.60 ± 0.42 MPa to 4.66 ± 0.26 MPa and 5.39 ± 0.11 MPa, respectively. This was because of the addition of rigid rod-like nanofillers in SPI films [21]. TCNCs showed an even better reinforcing effect compared with HCNCs. The tensile strength and Young’s modulus of TCNC-film-20% were 8.81 ± 0.18 MPa and 4.02 ± 0.34 MPa, which were 3.39- and 9.80-fold of those of SPI film (2.60 ± 0.42 MPa and 0.41 ± 0.12 MPa), respectively. This was because of the higher aspect ratio and better dispersion of TCNCs in water and SPI film, leading to a faster and uniform diffusion of stress [54]. On the other hand, the elongation at break of SPI/CNC films presented a significant decrease with the addition of CNCs, because the reinforcement restricted the mobility of protein molecules [55]. It was worth noting that the different CNC contents led to a significantly increased tensile strength (*p* < 0.05), but the change in elongation at break was not obvious (*p* > 0.05).

## 4. Conclusions

Two kinds of CNCs with different functional groups were successfully extracted from textile waste through sulfuric acid hydrolysis and three-step oxidization. TEM and XRD results revealed that both HCNCs and TCNCs had the rod-like shape and cellulose *I_β_* crystalline structure, with a high crystallinity index of 89.88% and 89.05%, respectively. A relatively higher aspect ratio was observed for TCNCs (17.10 ± 12.85) compared with HCNCs (10.00 ± 3.39). Those results demonstrated that textile waste materials offer a potential feedstock for the extraction of CNCs. Their application in reinforcing SPI film was investigated and summarized in Table 1. The SPI/CNC films maintained the high transparency with the addition of 20% CNCs. Besides, the WVP of HCNC-film-20% and TCNC-film-20% presented a significant decrease by around 29%. The mechanical property tests indicated that the tensile strength and Young’s modulus of the composite films were remarkably improved. When adding 20% CNCs, the tensile strength of HCNC-film and TCNC-film was 2.07-fold and 3.39-fold higher than that of SPI film, and their Young’s modulus showed 1.73-fold and 8.80-fold increases. This work reported the possibilities of directly extracting CNCs from textile waste, contributing to solving the cellulosic waste disposal problem, and the derived CNCs showed significant potential in food packaging application.

## Figures and Tables

**Table 1:** Physical properties of soy protein isolate (SPI) and SPI/cellulose nanocrystal (CNC) films. WVP, water vapor permeability.

Samples	Tensile Strength (MPa)	Elongation at Break (%)	Young’s Modulus (MPa)	WVP (10^−6^ g m^−1^ h^−1^ Pa^−1^)	Transmittance (%, 600 nm)
SPI film	2.6 ± 0.42	172.15 ± 16.69	0.41 ± 0.12	1.62 ± 0.15	48.7
HCNC-film-10%	4.66 ± 0.26	95.74 ± 3.93	1.04 ± 0.10	1.35 ± 0.02	40.9
HCNC-film-20%	5.39 ± 0.11	69.69 ± 4.80	1.12 ± 0.04	1.15 ± 0.11	37.1
TCNC-film-10%	7.11 ± 0.35	54.06 ± 15.27	1.66 ± 0.09	1.25 ± 0.02	37.0
TCNC-film-20%	8.81 ± 0.18	25.37 ± 5.77	4.02 ± 0.34	1.15 ± 0.02	34.1
